# Influencing factors of different metabolic status in hospitalized patients with schizophrenia

**DOI:** 10.3389/fpsyt.2024.1436142

**Published:** 2024-07-18

**Authors:** Mubing Ding, Shaotong Zhang, Zaochen Zhu, Renliang Cai, Jin Fang, Chao Zhou, Xiangrong Zhang, Xinyu Fang

**Affiliations:** ^1^ Department of Psychiatry, Beijing Anding Hospital Affiliated to Capital Medical University Wuhu Hospital & Wuhu Fourth People’s Hospital, Wuhu, China; ^2^ Department of Psychiatry, The Affiliated Brain Hospital of Nanjing Medical University, Nanjing, China

**Keywords:** schizophrenia, obesity, metabolism, risk factors, glucose, lipid

## Abstract

**Objective:**

The aim of this study was to explore the risk factors for different metabolic status in patients with schizophrenia.

**Methods:**

A total of 968 hospitalized patients with schizophrenia were recruited. Fasting blood glucose (GLU) and lipid profile, including total cholesterol (TC), low-density lipoprotein cholesterol (LDL-C), high-density lipoprotein cholesterol (HDL-C), and triglyceride (TG) were measured. Schizophrenia patients were divided into four groups: normal metabolism and weight (NMNW), abnormal metabolism and normal weight (AMNW), normal metabolism and overweight/obesity (NMO), and abnormal metabolism and overweight/obesity (AMO).

**Results:**

Our results showed that NMNW, AMNW, NMO, and AMO accounted for 25.3%, 12.7%, 25.4%, and 36.6%, respectively. There were significant differences in age, disease duration, body mass index (BMI), waist circumference, chronic disease, systolic blood pressure (SBP), diastolic blood pressure (DBP), GLU, TG, TC, HDL-C, and LDL-C among these four groups (all p < 0.05). With the NMNW group as the reference, the disordered multiple classification regression analysis showed that chronic disease was a significant risk factor for AMNW (OR = 5.271, 95% CI = 3.165 to 8.780, p < 0.001) and AMO (OR = 3.245, 95% CI = 2.004 to 5.254, p < 0.001), age was an important protective factor for NMO (OR = 0.968, 95% CI = 0.943 to 0.994, p = 0.015) and AMO (OR = 0.973, 95% CI = 0.948 to 0.999, p < 0.042), waist circumference was a significant risk factor for NMO (OR = 1.218, 95% CI = 1.180 to 1.257, p < 0.001) and AMO (OR = 1.252, 95% CI = 1.212 to 1.291, p < 0.001), and college education was an obvious protective factor for AMO (OR = 0.343, 95% CI = 0.123 to 0.953, p < 0.040) among patients with schizophrenia.

**Conclusion:**

The findings of our study underscored the importance of factors such as age, education level, chronic disease, and waist circumference when exploring the influencing factors and biological mechanisms of obesity-related metabolic problems in schizophrenia patients.

## 1 Introduction

Schizophrenia is a psychiatric disorder characterized by distorted thinking, perception, and emotional disharmony (1). It affects approximately 1% of the world population, with a heightened risk reaching 2.5 times for mortality compared to that in the general population (2), consequently leading to 10–20 years of life expectancy lost in patients with schizophrenia (3). Premature mortality is primarily attributed to preventable physical illness, especially cardiovascular disease (4). Large-scale epidemiological investigations have consistently shown that cardiovascular disease is the leading cause of death in individuals with schizophrenia, accounting for up to one-third of all cases (4, 5).

Patients diagnosed with schizophrenia are associated with a range of unfavorable cardiovascular traits, such as a higher risk of abdominal obesity, higher blood pressure, higher risk of hyperlipidemia, hyperglycemia, and insulin resistance (6). The occurrence of these metabolic disorders may be attributed to multiple factors, such as genetic liability, psychotropic drugs used, poor eating habits, and lifestyle (7, 8). A recent meta-analysis demonstrated that schizophrenia patients had an elevated risk of metabolic syndrome, even in first-episode drug-naïve patients, compared to the age- and gender-matched controls (9). Additionally, a Mendelian randomization study has indicated that schizophrenia was genetically associated with dyslipidemia (10). Moreover, a previous study has reported that schizophrenia patients receiving olanzapine monotherapy for over 2 years had a 44% risk of developing metabolic syndrome (11). Unhealthy habits such as poor diet, smoking, and lack of exercise are prevalent among schizophrenia patients, which were constantly shown to increase the risk of developing metabolic disorders (12). Taken together, patients with schizophrenia had an increased risk of metabolic disorders and cardiovascular disease due to various intrinsic and extrinsic factors, ultimately exacerbating the mortality outcome. Hence, knowing the risk factors for metabolic disorders in patients diagnosed with schizophrenia can help early cardiovascular disease screening and treatments and eventually improve their outcomes.

As we know, metabolic disorders encompass abnormalities in body mass, blood glucose, blood pressure, blood lipids, and other related indicators (13). Intriguingly, patients with schizophrenia do not usually have the above metabolic indicator abnormality simultaneity; some may only demonstrate alterations in one or two parameters of body mass, blood glucose, blood pressure, or blood lipids. However, most previous studies have only focused on one aspect of metabolic indicators and compared the differences between schizophrenia patients with and without obesity (14, 15), with and without diabetes (16), or with and without hyperlipidemia (17, 18). To better understand this topic, we divided schizophrenia patients into four groups: patients with normal metabolism and weight (NMNW), patients with abnormal metabolism and normal weight (AMNW), patients with normal metabolism and overweight/obesity (NMO), and patients with abnormal metabolism and overweight/obesity (AMO). We aimed to compare the basic data differences between groups, thus providing directions regarding more targeted supervision and intervention of these patients.

## 2 Methods

### 2.1 Participants

A total of 968 schizophrenia patients were recruited from the hospitalization department of Wuhu Fourth People’s Hospital, China. All patients met the following criteria: 1) diagnosed with schizophrenia according to the Diagnostic and Statistical Manual of Mental Disorders, Fifth Edition (DSM-5), using modified sections of the Structured Clinical Interview for DSM-5 disorders (SCID) by at least two experienced psychiatrists; 2) age range from 18 to 65 years and Han Chinese ethnicity; and 3) body mass index (BMI) ≥18.5 kg/m^2^ (calculated by weight divided by the square of height). The exclusion criteria included the following: 1) any other major Axis disorder, 2) pregnant or breastfeeding, and 3) substance (except for tobacco) abuse/dependence.

The study was approved by the Institutional Review Board of Wuhu Fourth People’s Hospital, and all participants were recruited between January 2023 and August 2023. Written informed consent was provided by all patients and/or their guardians after the research process was fully explained.

### 2.2 Anthropometric and metabolic parameters

A questionnaire was applied to collect the basic demographic and clinical data, including age, sex, height, weight, education level, marital status, smoking and drinking habits, chronic physical disease (excluding metabolic diseases directly related to blood pressure, blood glucose, and blood lipids, but including respiratory system diseases, digestive system diseases, etc.), and disease duration. Blood samples from the participants were obtained following an overnight fasting period. The serum was separated, aliquoted, and stored at −80°C until used for the assay. Fasting blood glucose (GLU) and lipid profile, including total cholesterol (TC), low-density lipoprotein cholesterol (LDL-C), high-density lipoprotein cholesterol (HDL-C), and triglyceride (TG), were measured using an automatic Biochemical Analyzer (HITACHI 7170A, Hitachi, Ltd., Tokyo, Japan). Blood pressure including systolic blood pressure (SBP) and diastolic blood pressure (DBP) was measured using an automated blood pressure machine before the blood was obtained. The waist circumference of patients was measured between the lower rib margin and the iliac crest after a normal expiratory breath (13).

In the present study, four groups: normal metabolism and weight (NMNW), abnormal metabolism and normal weight (AMNM), normal metabolism and overweight/obesity (NMO), and abnormal metabolism and overweight/obesity (AMO), according to their BMI and metabolic parameters. Overweight/obesity was defined as BMI ≥ 24 kg/m^2^, and normal weight was defined as a BMI between 18.5 and 24 kg/m^2^ (14). According to the definition by the National Cholesterol Education Program Adult Treatment Panel III (NCEP ATP III), abnormal metabolism was diagnosed using any two of the following criteria: 1) blood pressure ≥130/85 mmHg, a history of hypertension, or received antihypertensive treatment; 2) fasting triglyceride level ≥1.7 mmol/L; 3) HDL-C level <1.03 mmol/L for men and <1.30 mmol/L for women; or 4) a fasting plasma glucose (FPG) level ≥5.6 mmol/L or has reported previously physician-diagnosed diabetes (19).

### 2.3 Statistical analysis

The data analysis was performed using IBM SPSS software (version 23.0, Chicago, IL, USA). The analysis of variance (ANOVA) or the chi-squared tests were used to compare the differences between groups, as appropriate. The Bonferroni correction was further used for multiple tests. Then, the disordered multiple classification regression analysis was conducted to identify risk factors related to abnormal metabolism and overweight/obesity, with all observed factors as independent variables and the four groups set as dependent variables (with normal metabolism and weight as the reference). All statistical tests were two-tailed, and statistical significance was set at alpha ≤ 0.05.

## 3 Results

Among 968 schizophrenia patients we included in the present study, 245 were NMNW (25.3%), 123 were AMNW (12.7%), 246 were NMO (25.4%), and 354 were AMO (36.6%). As shown in **Table 1**, there were significant differences in age, disease duration, BMI, waist circumference, chronic disease, SBP, DBP, GLU, TG, TC, HDL-C, and LDL-C among these four groups (all p < 0.05). After the Bonferroni correction was conducted, we found that schizophrenia patients with AMNW and AMO were older than patients with NMNW and NMO (Bonferroni-corrected p < 0.05), while there was no significant difference in age between patients with AMNW and AMO (Bonferroni-corrected p > 0.05) and between patients with NMNW and NMO (Bonferroni-corrected p > 0.05). Patients with AMNW had a longer disease duration compared to patients with NMO after the Bonferroni correction was conducted (Bonferroni-corrected p = 0.044), but no difference among other groups remained (Bonferroni-corrected p > 0.05). After the Bonferroni correction was conducted, there were significant differences in BMI and waist circumference among groups (Bonferroni-corrected p < 0.001) except for the two groups of NMNW and AMNW (Bonferroni-corrected p > 0.05). For SBP, DBP, and GLU, our results showed that patients with AMNW and AMO had a higher level of these three indicators than patients with NMNW and NMO (Bonferroni-corrected p < 0.001), while there was no significant difference between patients with AMNW and AMO (Bonferroni-corrected p > 0.05) and between patients with NMNW and NMO (Bonferroni-corrected p > 0.05). The difference in TG among the four groups still remained after the Bonferroni correction was conducted (Bonferroni-corrected p < 0.05), with NMNW having the lowest TG level, followed by NMO and then AMNW, and AMO having the highest level. After the Bonferroni correction was conducted, the difference in HDL-C levels between groups (Bonferroni-corrected p < 0.001) was still significant except for the two groups of AMNW and AMO (Bonferroni-corrected p > 0.05), and the differences in LDL-C and TC levels between the NMNW and NMO groups, between the NMNW and AMO groups, between the AMNW and AMO groups, and between the NMO and AMO groups still remained after the Bonferroni correction was conducted (Bonferroni-corrected p < 0.05), but there were no differences in LDL-C and TC levels between the NMNW and AMNW groups and between the AMNW and NMO groups after the Bonferroni correction was conducted (Bonferroni-corrected p > 0.05). Additionally, we found that schizophrenia patients with AMNW and AMO had a higher rate of chronic disease compared to the patients with NMNW and NMO (Bonferroni-corrected p < 0.05), while there was no significant difference in age between patients with AMNW and AMO (Bonferroni-corrected p > 0.05) and between patients with NMNW and NMO (Bonferroni-corrected p > 0.05) after the Bonferroni correction was conducted.

**Table 1:** Comparisons among four different patient groups.

	NMNW (N = 245)	AMNW (N = 123)	NMO (N = 246)	AMO (N = 354)	F/x^2^	p
Age	44.61 ± 11.96 a	49.81 ± 9.21 b	43.81 ± 11.06 a	47.03 ± 9.82 b	11.217	<0.001
Disease duration	16.55 ± 10.10 a,b	19.08 ± 10.76 a	16.24 ± 8.83 b	18.11 ± 9.25 a,b	3.757	0.011
BMI	21.20 ± 1.39 a	21.63 ± 1.31 a	26.47 ± 2.33 b	27.53 ± 2.80 c	519.779	<0.001
Waist	82.52 ± 7.07 a	83.15 ± 7.23 a	93.60 ± 8.47 b	96.48 ± 8.31 c	197.583	<0.001
SBP	117.13 ± 10.85 a	121.63 ± 9.99 b	118.01 ± 7.29 a	121.34 ± 10.92 b	12.316	<0.001
DBP	75.24 ± 6.96 a	78.24 ± 6.87 b	75.40 ± 6.13 a	78.06 ± 7.52 b	12.929	<0.001
GLU	4.61 ± 0.75 a	5.20 ± 1.26 b	4.66 ± 0.50 a	5.48 ± 1.49 b	41.870	<0.001
TG	1.02 ± 0.49 a	1.66 ± 0.85 b	1.24 ± 0.43 c	2.15 ± 1.18 d	103.203	<0.001
HDL	1.16 ± 0.29 a	0.93 ± 0.18 b	1.06 ± 0.27 c	0.90 ± 0.19 b	66.111	<0.001
LDL	2.38 ± 0.65 a	2.54 ± 0.70 a,b	2.69 ± 0.67 b	2.99 ± 0.81 c	37.000	<0.001
TC	4.03 ± 0.79 a	4.09 ± 0.94 a,b	4.26 ± 0.84 b	4.62 ± 0.99 c	24.806	<0.001
Age range					28.468	<0.001
≥60	15 (6.1%) a	11 (8.9%) a	12 (4.9%) a	21 (5.9%) a		
40–59	152 (62.0%) a	98 (79.7%) b	151 (61.4%) a	253 (71.5%) a,b		
18–39	78 (31.8%) a,b	14 (11.4%) c	83 (33.7%) b	80 (22.6%) a		
Sex					0.469	0.926
Male	173 (70.6%)	88 (71.5%)	179 (72.8%)	249 (70.3%)		
Female	72 (29.4%)	35 (28.5%)	67 (27.2%)	105 (29.7%)		
Education					20.113	0.065
Illiteracy	21 (8.6%)	16 (13.0%)	21 (8.5%)	33 (9.3%)		
Elementary school	48 (19.6%)	31 (25.2%)	65 (26.4%)	111 (31.4%)		
Junior high school	108 (44.1%)	43 (35.0%)	106 (43.1%)	128 (36.2%)		
Senior high school	42 (17.1%)	25 (20.3%)	36 (14.6%)	62 (17.5%)		
College and above	26 (10.6%)	8 (6.59%)	18 (7.3%)	20 (5.6%)		
Marital status					9.467	0.149
Unmarried	147 (60.0%)	69 (56.1%)	144 (58.5%)	197 (55.6%)		
Married	50 (20.4%)	29 (23.6%)	64 (26.0%)	106 (29.9%)		
Divorced or widowed	48 (19.6%)	25 (20.3%)	38 (15.4%)	51 (14.4%)		
Smoking					1.655	0.647
No	196 (80.0%)	95 (77.2%)	188 (76.4%)	284 (80.2%)		
Yes	49 (20.0%)	28 (22.8%)	58 (23.6%)	70 (19.8%)		
Drinking					5.068	0.167
No	237 (96.6%)	113 (91.9%)	237 (96.3%)	337 (95.2%)		
Yes	8 (3.3%)	10 (8.1%)	9 (3.7%)	17 (4.8%)		
Chronic disease					138.516	<0.001
No	198 (80.8%) a	50 (40.7%) b	202 (82.1%) a	166 (46.9%) b		
Yes	47 (19.2%) a	73 (59.3%) b	44 (17.9%) a	188 (53.1%) b		

The lowercase letters (a, b, c, and d) reflect the results of multiple corrections (Bonferroni correction) across groups, with identical letters indicating no statistical difference and different letters indicating a statistical difference between columns.

NMNW, normal metabolism and weight; AMNW, abnormal metabolism and normal weight; NMO, normal metabolism and overweight/obesity; AMO, abnormal metabolism and overweight/obesity; GLU, fasting blood glucose; TC, total cholesterol; LDL-C, low-density lipoprotein cholesterol; HDL-C, high-density lipoprotein cholesterol; TG, triglyceride; SBP, systolic blood pressure; DBP, diastolic blood pressure.

With the NMNW group as the reference, our disordered multiple classification regression analysis showed that the chronic disease was a significant risk factor for AMNW (OR = 5.271, 95% CI = 3.165 to 8.780, p < 0.001) and AMO (OR = 3.245, 95% CI = 2.004 to 5.254, p < 0.001), age was an important protective factor for NMO (OR = 0.968, 95% CI = 0.943 to 0.994, p = 0.015) and AMO (OR = 0.973, 95% CI = 0.948 to 0.999, p < 0.042), waist circumference was a significant risk factor for NMO (OR = 1.218, 95% CI = 1.180 to 1.257, p < 0.001) and AMO (OR = 1.252, 95% CI = 1.212 to 1.291, p < 0.001), and college education was an obvious protective factor for AMO (OR = 0.343, 95% CI = 0.123 to 0.953, p < 0.040) among patients with schizophrenia (see **Table 2**).

**Table 2:** The results of disordered multiple classification regression analysis.

	AMNW			NMO			AMO		
	Exp(B)	95% CI	p	Exp(B)	95% CI	p	Exp(B)	95% CI	p
Age	1.018	0.989–1.049	0.226	0.968	0.943–0.994	0.015	0.973	0.948–0.999	0.042
Disease duration	1.007	0.978–1.035	0.652	1.020	0.992–1.050	0.164	1.027	0.999–1.056	0.055
Abdominal Cir	1.000	0.967–1.033	0.987	1.218	1.180–1.257	<0.001	1.252	1.212–1.291	<0.001
Sex									
Male	1.00			1.00			1.00		
Female	0.918	0.502–1.679	0.929	1.060	0.611–1.839	0.836	1.061	0.616–1.829	0.830
Education									
Illiteracy	1.00			1.00			1.00		
Elementary school	1.086	0.456–2.586	0.852	1.065	0.458–2.474	0.884	1.228	0.545–2.769	0.620
Junior high school	0.800	0.341–1.875	0.607	0.719	0.316–1.635	0.431	0.630	0.284–1.401	0.257
Senior high school	1.163	0.462–2.924	0.749	0.635	0.252–1.597	0.334	0.768	0.316–1.868	0.561
College and above	0.800	0.251–2.549	0.706	0.385	0.138–1.073	0.068	0.343	0.123–0.953	0.040
Marital status									
Unmarried	1.00			1.00			1.00		
Married	1.032	0.548–1.945	0.921	1.525	0.457–1.529	0.147	1.432	0.814–2.516	0.213
Divorced or widowed	0.873	0.454–1.677	0.683	0.836	0.863–2.697	0.560	0.647	0.353–1.185	0.159
Smoking									
No	1.00			1.00			1.00		
Yes	0.893	0.465–1.717	0.735	1.615	0.923–2.825	0.093	1.198	0.677–2.120	0.536
Drinking									
No	1.00			1.00			1.00		
Yes	2.188	0.712–6.722	0.172	0.630	0.201–1.978	0.429	1.104	0.378–3.227	0.857
Chronic disease									
No	1.00			1.00			1.00		
Yes	5.271	3.165–8.780	<0.001	0.690	0.403–1.180	0.175	3.245	2.004–5.254	<0.001

NMNW, normal metabolism and weight; AMNW, abnormal metabolism and normal weight; NMO, normal metabolism and overweight/obesity; AMO, abnormal metabolism and overweight/obesity.

## 4 Discussion

In the present study, a preliminary comparison was made among schizophrenia patients with different BMI and metabolic status, and the influencing factors of different metabolic status in those patients were further explored. Our results showed that NMNW, AMNW, NMO, and AMO accounted for 25.3%, 12.7%, 25.4%, and 36.6% respectively. In addition to BMI, SBP, DBP, GLU, TG, TC, HDL-C, and LDL-C, there were also significant differences in age, disease duration, waist circumference, and chronic disease among four patient groups. Further disordered multiple classification regression analysis demonstrated that chronic disease was a significant risk factor for AMNW and AMO, waist circumference was a significant risk factor for NMO and AMO, age was an important protective factor for NMO and AMO, and college education was an obvious protect factor for AMO in patients with schizophrenia.

A newly published meta-analysis revealed a combined pooled prevalence of overweight and obesity at 58.6% among patients with schizophrenia (20), a finding consistent with our current study (25.4% + 36.6%). Furthermore, an earlier meta-analysis comprising 77 publications with a sample size of 25,692 schizophrenia patients indicated a prevalence of metabolic syndrome in this population at 32.5% (21). Previous research has also shown that the incidence of metabolic syndrome varies in first-episode drug-naïve schizophrenia patients and those undergoing long-term atypical antipsychotic-treated schizophrenia patients ranging from 19% to 44% (11, 22). In the current study, the prevalence of metabolic syndrome ranged from at least 36.6% to a maximum of 49.3% among chronic hospitalized schizophrenia. Collectively, this body of evidence supports that schizophrenia patients have a heightened prevalence of overweight/obesity and metabolic abnormalities. However, not all schizophrenia patients with metabolic syndrome have overweight/obesity, and not all obese patients have metabolic syndrome, so the heterogeneity of metabolic abnormalities should be considered when exploring the association between metabolic status and demographic, clinical, or even neurobiological parameters in schizophrenia patients. Hence, we carried out this preliminary investigation.

There was a significant difference in age among the four patient groups; furthermore, regression analysis revealed that age was a protective factor for overweight/obesity (regardless of metabolism) in schizophrenia patients. This was different from a recent study conducted in China, which found that age was a risk factor for AMNW and NMO when compared to NMNW in schizophrenia (23). This discrepancy may be caused by the age distribution of the patients recruited in the different studies. In the present study, schizophrenia patients aged 18–39 years, 40–59 years, and over 60 years accounted for 26.3%, 67.6%, and 6.1%, respectively, but in that recent study, patients in the corresponding age groups accounted for 45.2%, 41.5%, and 13.3%, respectively. As we know, many functions of the body are degraded as age slowly increases; for example, digestive ability is not as good as before, thus leading to weight loss in older patients. Additionally, it should be pointed out that age may also reflect the length of the disease course, which together affects metabolic status in patients with schizophrenia; although our regression analysis did not find a direct effect of disease duration on metabolic status, the protective effect of age needs to be interpreted with caution. Therefore, future studies should enlarge the sample size and recruit schizophrenia patients of different ages in balance to further rigorously explore the effects of age and disease duration on body weight and metabolism.

Moreover, our findings revealed variations in waist circumference among schizophrenia patients with different metabolic status, with waist circumference proving to be a significant risk for overweight/obesity (NMO and AMO) in this population. This was also supported by a recent study that indicated that waist circumference was a prominent risk factor for obesity in schizophrenia patients (24). Interestingly, a longitudinal study demonstrated a significant increase in waist circumference following 6 months of antipsychotic treatment (25), suggesting its potential utility in the early monitoring of drug-induced weight and associated metabolic disorders among schizophrenia patients. In addition, waist circumference is widely regarded as a reliable indicator of abdominal adiposity and a valuable predictor of cardiovascular disease risk, particularly in situations where the use of radiological imaging is not feasible (26, 27).

We have also discovered that college education is an important protective factor for AMO, although it has no significant effect on AMNW and NMO in patients with schizophrenia. This means that high educational attainment has some protective effect against concurrent overweight/obesity and metabolic abnormalities in schizophrenia patients. The possible explanation is that highly educated individuals tend to show higher self-discipline and pay more attention to weight control, diet control, and physical exercise, especially when the body experiences weight changes or metabolic abnormalities, and these individuals are more alert to avoid developing a more serious metabolic state. This explanation needs to be further verified by future studies (28).

As we know, obesity and metabolic abnormalities are common chronic diseases in humans. In this study, we further analyzed the effects of chronic diseases other than obesity and glycolipid metabolism on obesity-related metabolic status in schizophrenia patients. Our findings revealed that the prevalence of chronic disease in schizophrenia patients with abnormal metabolism (AMNW or AMO) was significantly higher than that in patients with normal metabolism (NMNW or NMO). Regression analysis further indicated that chronic diseases were a risk factor for abnormal metabolism in patients with schizophrenia. A recent observational multicohort study also demonstrated that obesity is highly correlated with 21 diseases, and the interconnectedness of multiple diseases accelerates the development of obesity-related morbidity (29). Research from China additionally supported the link between obesity and the risk of chronic diseases (30). Overall, the aforementioned evidence supports the notion that obesity and other chronic diseases are interconnected, underscoring the need for future studies to consider and address the influence of other chronic diseases when investigating the influencing factors and biological mechanisms behind obesity-related metabolic issues in patients with schizophrenia patients. Furthermore, knowing what drives these associations is crucial to increasing the quality and duration of life for those patients.

Several limitations should be mentioned here. First, with a cross-sectional study design, we were unable to make a causal relationship between different metabolic status and factors in patients with schizophrenia. Second, only a few factors were investigated in the present study, while other information related to metabolic syndrome, such as physical activity and dietary habits, was not recorded. In addition, it is well known that antipsychotic drugs can have serious metabolic side effects but vary between drugs (31, 32). However, the present study did not consider and calculate the effects of the duration, types, and dosage of antipsychotic drugs on the metabolic status of schizophrenia patients. Third, all patients were of chronically hospitalized types and with long-term medications treated in one hospital, which limits the generalizability of our findings. Therefore, future studies based on longitudinal design and incorporating more relevant factors are warranted to explore the influence factors and even biological mechanism of different BMI and metabolic status in schizophrenia patients.

In summary, our study demonstrated that age, disease duration, waist circumference, and chronic disease are important influencing factors in overweight/obesity and metabolic abnormalities in schizophrenia, thus underscoring the importance of strict monitoring and management of these factors to reduce the risk of obesity-related metabolic problems. Additionally, future neurobiological studies on this topic should also consider the influence of these fundamental factors.

## Data availability statement

The raw data supporting the conclusions of this article will be made available by the authors, without undue reservation.

## Ethics statement

The studies involving humans were approved by the Fourth People’s Hospital of Wuhu. The studies were conducted in accordance with the local legislation and institutional requirements. Written informed consent for participation in this study was provided by the participants’ legal guardians/next of kin.

## Author contributions

MD: Data curation, Formal analysis, Investigation, Methodology, Software, Writing – original draft, Resources, Visualization. SZ: Data curation, Formal analysis, Methodology, Software, Writing – original draft, Visualization. ZZ: Data curation, Formal analysis, Methodology, Software, Writing – original draft, Investigation, Resources, Visualization. RC: Data curation, Formal analysis, Methodology, Writing – original draft. JF: Data curation, Formal analysis, Methodology, Writing – original draft. CZ: Data curation, Formal analysis, Methodology, Writing – original draft. XZ: Conceptualization, Funding acquisition, Project administration, Supervision, Validation, Writing – review & editing. XF: Conceptualization, Funding acquisition, Project administration, Supervision, Validation, Writing – original draft, Writing – review & editing.
